# Gene expression profile of mitogen-activated kinases and microRNAs controlling their expression in HaCaT cell culture treated with lipopolysaccharide A and cyclosporine A

**DOI:** 10.1080/15384101.2024.2320508

**Published:** 2024-03-06

**Authors:** Michał Wójcik, Nikola Zmarzły, Alicja Derkacz, Tomasz Kulpok-Bagiński, Natasza Blek, Beniamin Oskar Grabarek

**Affiliations:** a Collegium Medicum, WSB University, Dabrowa Gornicza, Poland; b Faculty of Medicine, Uczelnia Medyczna im. Marii Skłodowskiej-Curie, Warszawa, Poland

**Keywords:** Mitogen-activated protein kinases, cyclosporine A, bacterial lipopolysaccharide A

## Abstract

Studies indicate that mitogen-activated protein kinases (MAPKs) are activated and overexpressed in psoriatic lesions. The aim of the study was to assess changes in the expression pattern of genes encoding MAPKs and microRNA (miRNA) molecules potentially regulating their expression in human adult low-calcium high-temperature (HaCaT) keratinocytes exposed to bacterial lipopolysaccharide A (LPS) and cyclosporine A (CsA). HaCaT cells were treated with 1 µg/mL LPS for 8 h, followed by treatment with 100 ng/mL cyclosporine A for 2, 8, or 24 h. Untreated cells served as controls. The molecular analysis consists of microarray, quantitative real-time polymerase chain reaction, and enzyme-linked immunosorbent assay analyses. The statistical analysis of the obtained results was performed using Transcriptome Analysis Console and STATISTICA 13.5 PL with the statistical significance threshold of *p* < 0.05. Changes in the expression profile of six mRNAs: dual-specificity phosphatase 1 (*DUSP1)*, dual-specificity phosphatase 4 (*DUSP4)*, mitogen-activated protein kinase kinase 2 (*MAP2K2)*, mitogen-activated protein kinase kinase 7 (*MAP2K7)*, mitogen-activated protein kinase kinase kinase 2 (*MAP3K2)* and mitogen-activated protein kinase 9 (*MAPK9)* in cell culture exposed to LPS or LPS and the drug compared to the control. We observed that under the LPS and cyclosporine treatment, the expression o/ miR-34a, miR-1275, miR-3188, and miR-382 changed significantly (*p* < 0.05). We demonstrated a potential relationship between *DUSP1* and miR-34a; *DUSP4* and miR-34a, miR-382, and miR-3188; *MAPK9* and miR-1275, *MAP2K7* and mir-200-5p; *MAP3K2* and mir-200-5p, which may be the subject of further research in the context of psoriasis.

## Introduction

Psoriasis is a chronic inflammatory autoimmune disease with various dermatological manifestations [1]. According to the clinical classification, the subtypes include psoriasis vulgaris, inverse psoriasis, guttate psoriasis, pustular psoriasis, and erythrodermic psoriasis. Psoriasis can also affect the joints, resulting in psoriatic arthritis [1]. In addition, nail psoriasis may develop, which affects more than half of psoriasis patients [1]. Significant progress in the treatment of psoriasis and the increased number of studies on this disease pose a challenge to both patients and doctors [2]. The etiology and pathogenesis of psoriasis are still not fully understood, which results in the lack of accurate therapeutic methods [2]. Difficulties in identifying the factors causing and exacerbating psoriasis result from complex interactions of the genetic background, environmental factors, and modulation of immunological processes [3]. During psoriasis exacerbations, clinical changes occur in the form of, among others, parakeratosis, infiltration of neutrophils, lymphocytes, and cytokines in both the epidermis and dermis. Skin changes result from an accelerated abnormal maturation cycle of keratinocytes, accompanied by increased inflammatory infiltration [2].

Regardless of the type of psoriasis, the appearance of the skin often causes discomfort [4]. Psoriasis is still a disease that cannot be completely cured, and patients are exposed to side effects of the medications they use [4]. The treatment aims to extend the remission period, alleviate symptoms, and improve the quality of life of psoriasis patients [4]. Drugs have local or generalized effects and their action is based on mechanisms that slow down the rate of keratinocyte metabolism, limit cell differentiation, and remove scales [5].

One of the drugs used is cyclosporine A (CsA), a strong immunosuppressive drug whose mechanism of action is related to inhibiting the proliferation of CD4^+^ lymphocytes, inhibiting the production of antibodies by B lymphocytes and cytokines, and stimulating suppressor lymphocytes [6]. According to European recommendations, CsA is recommended mainly for short-term treatment aimed at achieving quick improvement [6]. Therapy lasting longer than 3–6 months is usually not recommended [6]. However, it is possible to continue therapy in patients

with good clinical response for up to 2 years or longer [6]. Treatment with CsA for more than 2 years should be carefully monitored due to the increased risk of side effects, including the development of skin cancer [7]. According to the recommendations of the Polish Dermatological Society dated 2020, CsA is recommended for the treatment of moderate and severe cases of psoriasis [7,8].

Although the pathogenesis of psoriasis is not fully understood and the applied treatment attempts do not lead to the complete disappearance of symptoms, it is known that the development and course of the disease are influenced by many external and internal factors [9,10]. Mitogen-activated protein kinases (MAPKs) are involved in the molecular mechanisms of psoriasis [11]. They participate in transmitting signals into the cell interior through a cascade of reactions, resulting in the activation of a number of proteins. In this way, MAPKs-dependent signaling pathways exert a modulatory effect on the expression of transcription factors, protein biosynthesis, cell division, and differentiation, and also influence cell survival and apoptosis [12]. MAPKs can be divided into three groups: extracellular signal-regulated kinases (ERKs), c-Jun N-terminal kinases (JNKs), and p38 MAPKs [13]. There are two isoforms of ERK: ERK1 and ERK2. Molecular pathways involving ERK1/2 are involved in the regulation of the cell cycle, cell proliferation, and differentiation [14]. JNKs are referred to as stress-activated kinases. There are three known isoforms: JNK1 and JNK2, as well as JNK3, found only in the brain and heart [15]. Signaling pathways involving JNK are activated by stressors, e.g. ultraviolet radiation, and are involved in cell differentiation, apoptosis, angiogenesis, and migration [15]. There are 4 isoforms of p38 MAPKs: alpha (α), beta (β), gamma (γ), sigma (δ). They are involved in cell differentiation and apoptosis. p38 MAPKs are activated by stress factors and as a result of the action of inflammatory cytokines and growth factors [16].

Studies indicate that MAPKs are activated and overexpressed in psoriatic lesions [17]. Activating the JNK pathway in keratinocytes may regulate the production of inflammatory cytokines and thus influence the recruitment of immune cells [18]. It is particularly important in autoimmune arthritic conditions, mainly rheumatoid arthritis, ankylosing spondylitis, and psoriatic arthritis [19]. However, there is still a lack of precise diagnostic and prognostic markers for psoriasis.

The aim of the study was to assess changes in the expression pattern of genes encoding MAPKs and miRNA molecules potentially regulating their expression in human adult low-calcium high-temperature (HaCaT) keratinocytes exposed to bacterial lipopolysaccharide A (LPS) and CsA.

## Material and methods

### Keratinocyte cell culture

HaCaT culture was carried out in 25 cm^2^ culture vessels and incubated in a Direct Heat CO_2_ incubator (Thermo Fisher Scientific, Waltham, MA) at 37°C, 5% CO_2_. The culture medium was Dulbecco’s modified Eagle’s medium (DMEM; Sigma-Aldrich, St. Louis, MO, USA) supplemented with glucose (4500 mg/L, Sigma-Aldrich, St. Louis, MO, USA), 10% fetal bovine serum (FBS; Sigma-Aldrich, St. Louis, MO, USA), penicillin (100 U/mL; Sigma-Aldrich, St. Louis, MO, USA), streptomycin (100 mg/mL; Sigma-Aldrich, St. Louis, MO, USA), and glutamine (2 mm; Sigma-Aldrich, St. Louis, MO, USA).

In the first stage, in accordance with our earlier investigations, HaCaT cells were subjected to an 8-hour incubation with 1 μg/mL of LPS [20–23] to induce inflammation, and in the next-stage CsA was added at a concentration of 100 ng/mL for 2, 8, and 24 hours. Culture untreated with LPS and the drug was the control culture. The concentration of CsA was selected empirically, taking into account the average concentration of CsA in the serum of patients treated with it for psoriasis, the same as in our previous work [21,22].

### Total RiboNucleic Acid (RNA) extraction

Total RNA extraction from LPS- and CsA-exposed and control HaCaT cultures was performed using Trizol reagent (Invitrogen Life Technologies, Carlsbad, CA, USA; Catalog number: 15596026), following the manufacturer’s instructions. In the next step, the obtained RNA extracts were purified using RNeasy mini kit (QIAGEN, Hilden, Germany; Catalog number: 74104) and DNase I (Fermentas International Inc., Burlington, ON, Canada; Catalog number: 18047019). Total RNA extracts were adjusted to a volume of 100 μL by adding an appropriate amount of RNase-free water. Then, 350 μL of RLT buffer, containing guanidinium isothiocyanate, 3.5 μL of beta-mercaptoethanol, and 250 μL of 96% ethanol were added to the samples. The entire mixture was mixed thoroughly and then transferred to the columns. After centrifuging twice, 350 μL of RQ1 solution was applied to the columns and centrifuged again. In the next step, the extracts underwent DNase I digestion. A combination of 10 μL of DNase and 70 μL of RDD buffer was applied to each column, followed by the addition of 350 μL of RW1 solution after a 15-minute incubation at room temperature. After centrifugation, 500 μL of RPE solution was applied to the columns. After another round of centrifugation, the columns were placed in new tubes, 30 μL of RNase-free water was added, followed by an incubation at room temperature for 10 minutes. This procedure was repeated, applying 20 μL of RNase-free water. Purified RNA extracts were stored at −80°C until further analysis.

#### Qualitative and quantitative evaluation of RNA extracts

The extracts were then qualitatively assessed by 1% agarose gel electrophoresis with the addition of Simply Safe dye (EurX, Gdańsk, Poland). Electrophoretic separation was conducted using a Submini apparatus (Kucharczyk, Warsaw, Poland). Analyzing the electropherogram under UV transilluminator light, employing a computerized gel documentation system, two bands corresponding to the 28S rRNA and 18S rRNA fractions were observed. Meanwhile, the concentration and purity of the extracts were evaluated using a spectrophotometer (Nanodrop®, Thermo Fisher Scientific, Waltham, MA, USA). The RNA concentration was determined, assuming that 1.0 OD260 equals 40 μg of RNA in 1 ml of extract. An increase in absorbance at other wavelengths indicated sample contamination (230 nm – contamination with hydrocarbons and aromatic compounds, ethanol residues; 280 nm – proteins; 320 nm – degradation of genetic material in the sample). The purity of RNA isolates was assessed based on the A260/A280 absorbance ratio, which should be in the range of 1.80–2.00.

### Determination of the expression profile of MAPKs using oligonucleotide microarrays

The expression profile of genes encoding MAPKs was assessed with the HG-U133_A2 oligonucleotide microarrays (Affymetrix, Santa Clara, CA, USA) and GeneChip™ 3 IVT PLUS reagent kit (Affymetrix, Santa Clara, CA, USA; Catalog Number 902,416) in accordance with the manufacturer’s recommendations. Each reaction was performed in triplicate. The procedure included five main steps: 1) cDNA synthesis; 2) cRNA synthesis, labeling, and fragmentation; 3) hybridization of samples with probes present on the microarray plate; 4) reading the fluorescence signal using Affymetrix Gene Array Scanner 3000 7 G and Gene Chip® Command Console® Software (Affymetrix, Santa Clara, CA, USA); 5) data analysis. For microarray analysis, the synthesis of double-stranded complementary DNA (cDNA) was carried out using the GeneChip 30IVT Express kit (at 42°C for 2 h). Following this, the sample underwent incubation with 20 μL of Second Strand master mix for 1 h at 16°C, followed by another incubation for 10 min at 65°C. Subsequently, the sample was subjected to incubation with 30 µL of IVTMaster Mix for cDNA for 16 h at 40°C, facilitating the synthesis of biotinylated aRNAs. The resulting aRNAs were then fragmented using a matrix fragmentation buffer for 35 min at 94°C. The hybridization process was conducted using the GeneChip hybridization, wash, and stain kit. The products from different stages of transcriptome determination were quantitatively assessed using spectrophotometric measurements and qualitatively analyzed through 1% agarose gel electrophoresis with the addition of Simply Safe dye (EurX, Gdansk, Poland). Only samples meeting the qualitative and quantitative evaluation criteria specified by Affymetrix were deemed eligible for subsequent stages of analysis.

### Identification of miRnas potentially regulating MAP kinase gene expression using miRNA microarrays

Analysis of miRNAs that significantly change their expression in HaCaT culture exposed to LPS and CsA was performed using the GeneChip miRNA 2.0 arrays (Affymetrix, Santa Clara, CA, USA) in accordance with the manufacturer’s recommendations. To determine the potential impact of the selected miRNAs on the expression of MAPKs genes, the Targetscan database (http://www.targetscan.org/; accessed 2021 Apr 14) [24] and miRanda (http://mirdb.org; accessed 2021 Apr 14) were used [25]. A predicted target with a prediction score of >80 is most likely to be real; however, if the score is below 60, then one needs to exercise caution, and it is recommended to have other supporting evidence as well” [25,26].

### Reverse Transcription-quantitative Polymerase Chain Reaction (RT-qPCR)

The results of the microarray experiment were validated by RT-qPCR. RT-qPCR reaction was performed for the following genes: dual-specificity phosphatase one (*DUSP1*); dual-specificity phosphatase four (*DUSP4*); mitogen-activated protein kinase kinase 2 (*MAP2K2*); mitogen-activated protein kinase kinase 7 (*MAP2K7*); mitogen-activated protein kinase kinase kinase 2 (*MAP3K2*); mitogen-activated protein kinase 9 (*MAPK9*); beta actin (*ACTB*). The Sensi-Fast reagent kit (Bioline, London, England) and the primers listed in Table 1 were used. ACTB was used as an endogenous control.

**Table 1.** Nucleotide sequence of primers used in RT-qPCR.

mRNA	Starter	Sequence	Product size (bp)	Tm (°C)
*DUSP1*	Forward	5’- GGATACGAAGCGTTTTCGGC-3’	1531	85.5
	Reverse	5’-AGAGGTCGTAATGGGGCTCT-3’		
*DUSP4*	Forward	5’-GACCGGCAAAAATACACGGG-3’	520	91.6
	Reverse	5’-GAACCTAGGATGTAGCCCGC-3’		
*MAP2K2*	Forward	5’- AGCTGGAGGAGCTGGAACTTG-3’	817	89.5
	Reverse	5’- CTATCCATCCCGTGACCGC-3’		
*MAP2K7*	Forward	5’-GACAGTTTCCCTACAAGAAC-3’	146	82.5
	Reverse	5’-CCTGTGATCTTTAGTAAGGC-3’		
*MAP3K2*	Forward	5’-TCTGTTTTATCTTCTCAGGCCA-3’	958	80.5
	Reverse	5’- CCCTGGGTCCTTCTAGCTCT-3’		
*MAPK9*	Forward	5’-AGTCATCCTGGGTATGGGCT-3’	570	81.3
	Reverse	5’-GCGTTGCTACTTACTGCTGC-3’		
*ACTB*	Forward	5’-TCACCCACACTGTGCCCATCTACGA-3’	295	87.8
	Reverse	5’-CAGCGGAACCGCTCATTGCCAATGG-3’		

DUSP1, dual specificity phosphatase 1; DUSP4, dual specificity phosphatase 4; MAP2K2, mitogen-activated protein kinase kinase 2; MAP2K7, mitogen-activated protein kinase kinase 7; MAP3K2, mitogen-activated protein kinase kinase kinase 2; MAPK9, mitogen-activated protein kinase 9; ACTB, beta actin; bp, base pair; Tm, melting temperature.

Reverse transcription and PCR was performed in the same tube without changing the reaction mixture in a total volume of 50 μL. Simultaneously with the test samples, an RT-qPCR reaction for *ACTB* mRNA was performed to confirm the nativeness of the RNA extracts and to assess the correctness of the amplification run. Each reaction was performed in triplicate. The thermal profile of the reaction was as follows: 1) reverse transcription (45°C; 10 min); 2) initial denaturation (95°C; 2 min); 3) 40 cycles consisting of denaturation (95°C; 5 s), attaching the primers to the template (60°C; 10 s), primer elongation (72°C; 5 s). Results were shown by using the 2^−∆∆Ct^ method (Fold change equal to 1 is control; >1 overexpression; <1 silencing). PCR efficiency ranged from 90% to 110%.

**Figure 1.**
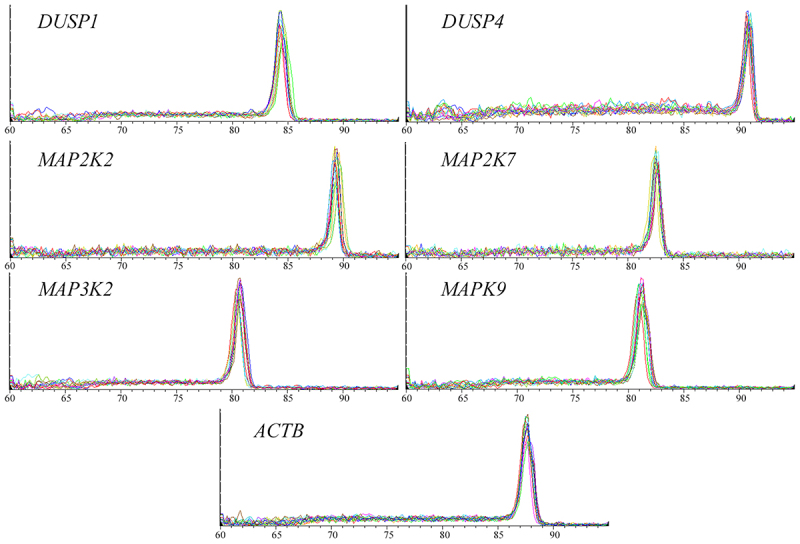
Examples of melting curves confirming the specificity of the reaction.

The specificity of the RT-qPCR reaction was confirmed by polyacrylamide gel electrophoresis and melting curve analysis. Examples of melting curves are shown in Figure 1.

DUSP1 – dual-specificity phosphatase 1; DUSP4 – dual-specificity phosphatase 4; MAP2K2 – mitogen-activated protein kinase kinase 2; MAP2K7 – mitogen-activated protein kinase kinase 7; MAP3K2 – mitogen-activated protein kinase kinase kinase 2; MAPK9 – mitogen-activated protein kinase 9; ACTB – beta actin.

### Determination of DUSP1, DUSP4, MAP2K2, MAP2K7, MAPK9 concentration by Enzyme-Linked Immunosorbent Assay (ELISA)

In the first step, the keratinocyte culture was washed with cold phosphate-buffered saline (PBS) and lysed in radioimmunoprecipitation assay lysis buffer (0.5% deoxycholate, 1% Nonidet *p*-40, 0.1% sodium dodecyl sulfate, 100 μg/mL phenylmethylsulfonyl fluoride, 1 mM Na2VO4 and 8.5 μg/mL aprotinin in PBS) with shaking for 20 min at 4°C, which allowed the extraction of total protein from the cells. Samples were then collected using a scraper and incubated for 60 min at 4°C, and then centrifuged for 15 min at 4°C. In the third step, the samples were transferred to the wells of an ELISA plate coated with biotin-labeled antibodies, washed and incubated with the avidin-horseradish peroxidase complex. A standard curve was prepared by plotting the average optical density and concentration of each standard and multiplying it by the dilution factor. Protein concentrations of DUSP1 (Cat no. MBS761028; MyBioSource, Inc. San Diego, CA, USA), DUSP4 (Cat. no. MBS2885922; MyBioSource, Inc. San Diego, CA, USA), MAP2K2 (Cat no MBS9331134; MyBioSource, Inc. San Diego, CA, USA), MAP2K7 (Cat no MBS643637; MyBioSource, Inc. San Diego, CA, USA), MAP3K2 (Cat no MBS9339652; MyBioSource, Inc. San Diego, CA, USA), and MAPK9 (USA; Cat no MBS1602553; MyBioSource, Inc. San Diego, CA, USA) were assessed by ELISA according to the manufacturer’s instructions. Each reaction was performed in triplicate.

### Statistical analysis

The statistical analysis of the obtained results was performed using Transcriptome Analysis Console (Thermo Fisher, USA) and STATISTICA 13.5 PL (StatSoft, Cracow, Poland) software with the statistical significance threshold of *p* < 0.05. The analysis included assessing the compliance of the obtained results with normal distribution using the Shapiro–Wilk test. Meeting the assumptions of normal distribution allowed for further analysis using parametric tests, i.e. a one-way analysis of variance (ANOVA) with Bonferroni correction test, preceded by the Levene’s test and followed by a Tukey’s post hoc test or Student’s t-test.

## Results

### Changes in MAP kinase mRNA expression obtained by microarray experiment

Based on the HARMONIZE database (www.maayanlab.cloud/Harmonizome; accessed 2022 Dec 29), 248 genes encoding proteins related to the MAPK pathway were selected [27]. At a further stage, the ANOVA with Bonferroni correction determined that 41 mRNAs differentiated the keratinocyte culture exposed to LPS and CsA from the control culture, and the number of mRNAs differentiating between each group was as follows: H_2 vs. C = 20 mRNAs, H_8 vs. C = 14 mRNAs, H_24 vs. C = 13 mRNAs, with 6 mRNAs differentiating the culture with the drug regardless of the incubation time: dual-specificity phosphatase 1 (*DUSP1)*, dual-specificity phosphatase 4 (*DUSP4)*, mitogen-activated protein kinase kinase 2 (*MAP2K2)*, mitogen-activated protein kinase kinase 7 (*MAP2K7)*, mitogen-activated protein kinase kinase kinase 2 (*MAP3K2)* and mitogen-activated protein kinase 9 (*MAPK9)*.

**Figure 2.**
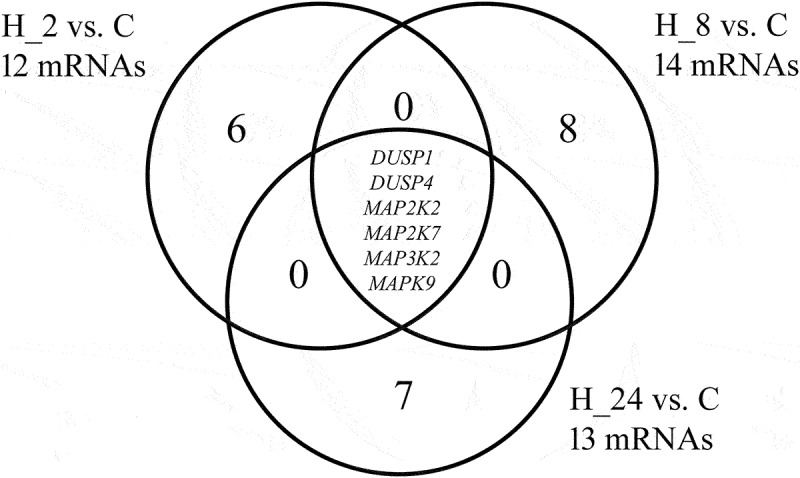
The Venn diagram of microarray results.

Changes in the expression profile of 6 mRNAs: *DUSP1*, *DUSP4*, *MAP2K2*, *MAP2K7*, *MAP3K2,* and *MAPK9* in cell culture exposed to LPS or LPS and the drug compared to the control are presented in Table 2. We noted that under the influence of LPS added to the culture, the expression of all transcripts was silenced. However, when CsA was added to the HaCaT culture, we found an increase in the transcriptional activity of 5 of 6 genes throughout the entire observation period. Only the expression of *DUSP4* after an 8-hour incubation of cells with the drug was silenced compared to the control, while prolonging the exposure time to the drug resulted in *DUSP4* expression at a level similar to the control culture (Table 2). Tukey’s post hoc test and Venn diagram revealed genes specific to a given incubation time of cells with LPS and drug or common to several groups (Figure 2).

**Table 2.** Expression pattern of MAPK-related genes in the HaCaT cell line exposed to LPS and then cyclosporine for 2, 8, and 24 hours compared to the control (*p* < 0.05).

ID	mRNA	LPS	LPS+CsA		
		log_2_ Fold Change			
		H_8 vs C	H-2 vs C	H-8 vs C	H-24 vs C
201041_s_at	*DUSP1*	(−)3.14 ± 0.34	(+)5.11 ± 0.37	(+)5.53 ± 0.55	(+)4.16 ± 0.13
201044_x_at		(−)3.11 ± 0.21	(+)6.88 ± 0.43	(+)5.55 ± 0.47	(+)4.22 ± 0.17
204014_at	*DUSP4*	(−)4.08 ± 0.43	(+)3.87 ± 0.24	(−)1.88 ± 0.11	(+)1.01 ± 0.18
204015_s_at		(−)4.09 ± 0.34	(+)2.12 ± 0.31	(−)2.09 ± 0.09	(−)1.01 ± 0.03
213490_s_at	*MAP2K2*	(−)3.51 ± 0.13	(+)2.80 ± 0.18	(+)4.08 ± 0.22	(+)3.90 ± 0.19
209952_s_at	*MAP2K7*	(−)4.70 ± 0.16	(+)2.31 ± 0.12	(+)2.17 ± 0.34	(+)3.50 ± 0.14
221695_s_at	*MAP3K2*	(−)4.09 ± 0.48	(+)4.01 ± 0.31	(+)3.47 ± 0.22	(+)3.51 ± 0.71
210570_x_at	*MAPK9*	(−)4.56 ± 0.31	(+)2.88 ± 0.21	(+)2.16 ± 0.22	(+)2.20 ± 0.31
203218_at		(−)4.43 ± 0.28	(+)2.71 ± 0.31	(+)2.19 ± 0.28	(+)2.12 ± 0.19

(+) – overexpression compared to the control; (−) – downregulated compared to the control; LPS – liposaccharide A; CsA – cyclosporine A; C – control culture; H_2, H_8, H_24 – culture exposed to cyclosporine A for 2, 8, 24 hours; DUSP1 – dual specificity phosphatase 1; DUSP4 – dual specificity phosphatase 4; MAP2K2 – mitogen-activated protein kinase kinase 2; MAP2K7 – mitogen-activated protein kinase kinase 7; MAP3K2 – mitogen-activated protein kinase kinase kinase 2; MAPK9 – mitogen-activated protein kinase 9.

C – control culture; H_2, H_8, H_24 – culture exposed to cyclosporine A for 2, 8, 24 hours; DUSP1 – dual-specificity phosphatase 1; DUSP4 – dual-specificity phosphatase 4; MAP2K2 – mitogen-activated protein kinase kinase 2; MAP2K7 – mitogen-activated protein kinase kinase 7; MAP3K2 – mitogen-activated protein kinase kinase kinase 2; MAPK9 – mitogen-activated protein kinase 9.

### Changes in MAP kinase mRNA expression obtained by RT-qPCR

**Figure 3.**
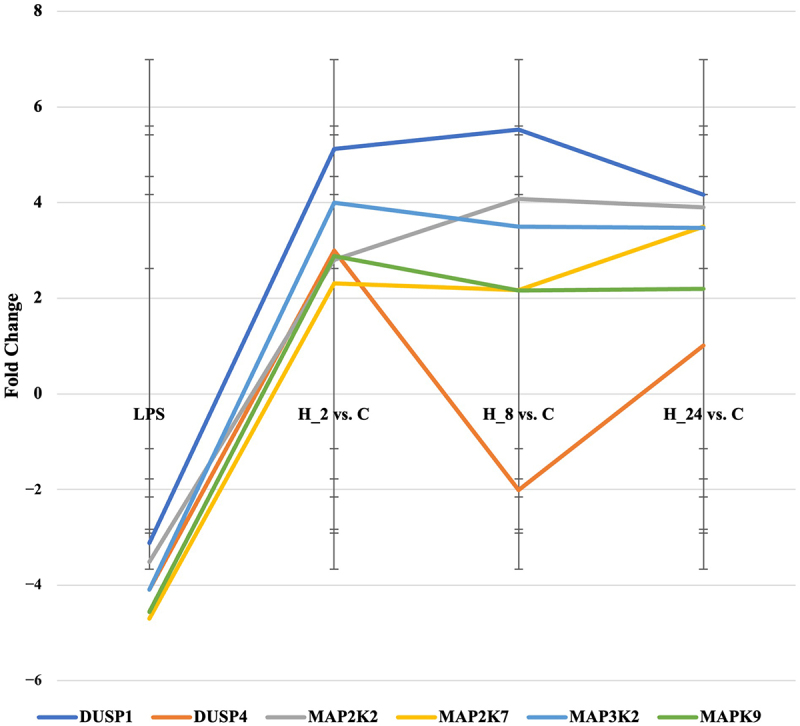
Expression profile of mRNA encoding selected MAPK-related genes in HaCaT treated with LPS and then with cyclosporine a for 2, 8, 24 hours compared to the control culture.

We validated the microarray experiment using RT-qPCR for six mRNAs that differentiated keratinocyte cultures from LPS and CsA regardless of the exposure time to the drug (Figure 3). We observed the same direction of expression change in both microarrays and RT-qPCR.

LPS – liposaccharide A; C – control culture; H_2, H_8, H_24 – culture exposed to cyclosporine A for 2, 8, 24 hours; DUSP1 – dual-specificity phosphatase 1; DUSP4 – dual-specificity phosphatase 4; MAP2K2 – mitogen-activated protein kinase kinase 2; MAP2K7 – mitogen-activated protein kinase kinase 7; MAP3K2 – mitogen-activated protein kinase kinase kinase 2; MAPK9 – mitogen-activated protein kinase 9.

In addition, in Table 3 we present the *p*-values for the ANOVA and Tukey’s post-hoc test. The analysis showed that the expression of all 6 mRNAs significantly increased in the culture treated with LPS and the addition of CsA compared to the culture with LPS only. This change was significant for all CsA treatment times.

**Table 3.** Results of ANOVA and Tukey’s post-hoc test for MAPK genes, the expression of which was determined by the qRT-PCR technique.

mRNA	*p*-value ANOVA	Adjusted *p*-value of Tukey’s post-hoc test					
		LPS vs. H_2	LPS vs. H_8	LPS vs. H_24	H_2 vs. H_8	H_2 vs. H_24	H_8 vs. H_24
*DUSP1*	<0.01	<0.001	<0.001	<0.001	0.504	<0.001	<0.001
*DUSP4*	<0.01	<0.001	<0.001	<0.001	<0.001	<0.001	<0.001
*MAP2K2*	<0.01	<0.001	<0.001	<0.001	<0.001	<0.001	0.040
*MAP2K7*	<0.01	<0.001	<0.001	<0.001	<0.001	<0.001	0.666
*MAP3K2*	<0.01	<0.001	<0.001	<0.001	<0.001	<0.001	0.900
*MAPK9*	<0.01	<0.001	<0.001	<0.001	<0.001	<0.001	0.900

LPS – lipopolysaccharide A; C – control culture; H_2, H_8, H_24 – culture exposed to cyclosporine A for 2, 8, 24 hours; DUSP1 – dual specificity phosphatase 1; DUSP4 – dual specificity phosphatase 4; MAP2K2 – mitogen-activated protein kinase kinase 2; MAP2K7 – mitogen-activated protein kinase kinase 7; MAP3K2 – mitogen-activated protein kinase kinase kinase 2; MAPK9 – mitogen-activated protein kinase 9.

In the case of LPS culture with CsA, the activity of *DUSP1*, *MAP3K2,* and *MAPK9* genes decreased significantly after 24 hours of exposure to CsA, in contrast to *MAP2K7*, the expression of which reached the highest level after 24 hours. In addition, *DUSP4* expression reached its lowest level after 8 hours of culture, and then began to increase significantly. In the case of *MAP2K2*, expression significantly increased after 8 hours of exposure to CsA, reaching a stable level.

### miRNA expression

In the next step, we determined which miRNAs differentiating the keratinocyte culture exposed to the drug compared to the control culture, taking into account the miRSVR parameter, potentially regulate the expression of *DUSP1*, *DUSP4*, *MAP2K2*, *MAP2K7*, *MAP3K2,* and *MAPK9*.

**Figure 4.**
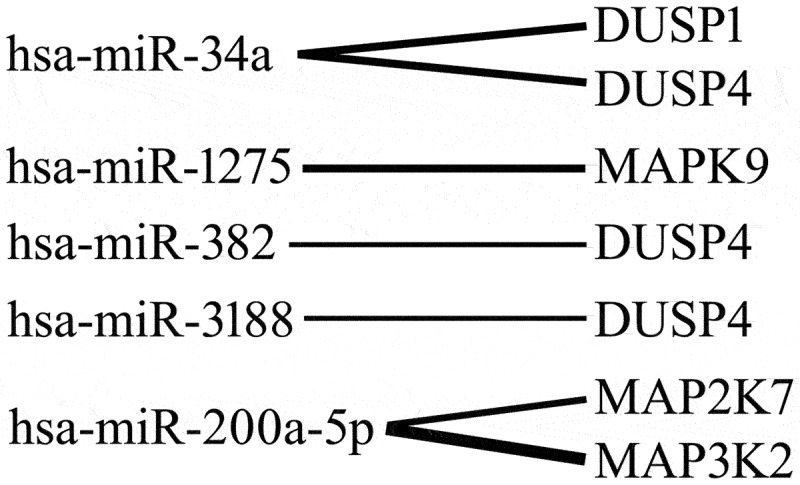
miRnas potentially modulating the expression of MAP kinase-related genes in keratinocytes exposed to cyclosporine a compared to the control.

DUSP1, dual-specificity phosphatase 1; DUSP4, dual-specificity phosphatase 4; MAP2K7, mitogen-activated protein kinase kinase 7; MAP3K2, mitogen-activated protein kinase kinase kinase 2; MAPK9, mitogen-activated protein kinase 9.

We demonstrated that miR-34a potentially regulates the activity of *DUSP1* and *DUSP4*. In turn, miR-1275 is involved in the regulation of *MAPK9* expression. Moreover, miR-382 and miR-3188 potentially regulate *DUSP4* levels, while mir-200-5p participates in the regulation of *MAP2K7* and *MAP3K2* levels. Predictive analysis showed no regulatory effect of miRNA molecules on *MAP2K2* expression (Figure 4).

In turn, Table 4 presents changes in the expression of selected miRNAs with statistical significance values obtained in the ANOVA and Tukey’s post-hoc tests. Only for miR-34a, there was no significant change in its expression under the influence of LPS or CsA added to cell culture. The level of miR-1275 increased significantly after the addition of CsA, reaching the highest activity after 24 hours. In the case of miR-3188, its expression significantly decreased after 2 hours, and prolonging the exposure time resulted in an increase in its level, but this was not a statistically significant change. Moreover, the expression of miR-382 and miR-200a-5p decreased significantly after the addition of CsA to the culture.

**Table 4.** Expression pattern of miRnas potentially regulating the expression of *DUSP1*, *DUSP4*, *MAP2K2*, *MAP2K7*, *MAP3K2* and *MAPK9* of the HaCaT cell line exposed to LPS and then cyclosporine for 2, 8, and 24 hours compared to the control.

miRNA	Expression					
	LPS vs. C	H-2 vs C	H-8 vs C	H-24 vs C	*p*-value ANOVA	*p*-value Tukey’s post-hoc test
miR-34a	(+)2.17 ± 0.32	(+)2.98 ± 0.21	(+)2.98 ± 0.18	(+)2.54 ± 0.22	0.6521	0.690^1^ 0.690^2^ 0.820^3^ 1^4^ 0.995^5^ 0.995^6^
miR-1275	(+)1.87 ± 0.43	(+)2.98 ± 0.11	(+)3.13 ± 0.18	(+)3.19 ± 0.21	<0.001	<0.001^1,2,3,4,5^ 0.514^6^
miR-3188	(+)1.99 ± 0.22	(+)1.22 ± 0.32	(+)2.10 ± 0.19	(+)2.19 ± 0.09	<0.001	<0.001^1,4,5^ 0.097^2^ 0.238^3^ 0.912^6^
miR-382	(+)4.98 ± 0.99	(+)2.19 ± 0.82	(+)2.88 ± 0.71	(+)2.18 ± 0.21	<0.001	<0.001^1,2,3^ 0.419^4^ 0.346^5^ 0.0376^6^
miR-200a-5p	(+)3.09 ± 0.21	(+)2.19 ± 0.34	(+)2.09 ± 0.12	(+)2.18 ± 0.54	<0.001	<0.001^1,2^ 0.036^3^ 0.419^4^ 0.900^5^ 0.031^6^

(+) – overexpression in comparison to the control; (−) – downregulated in comparison to the control; LPS – liposaccharide A; C – control culture; H_2, H_8, H_24 – culture exposed to cyclosporine A for 2, 8, 24 hours; statistically significant differences between:^1^LPS vs. H_2; ^2^LPS vs. H_8; ^3^LPS vs. H_24; ^4^H_2 vs. H_8; ^5^ H_2 vs. H_24; ^6^H_8 vs. H_24.

### Protein concentrations of DUSP1, DUSP4, MAP2K2, MAP2K7, MAPK9

The assessment of the concentration profile of selected proteins related to MAPK pathways showed that the addition of LPS to the HaCaT culture results in a decrease in the expression of each protein compared to the control culture (Table 5; *p* < 0.05). We also noted that when CsA was added to the cell culture, the concentrations of *DUSP1*, *DUSP4*, *MAP3K2*, and *MAPK9* were below the detection threshold. In turn, for *MAP2K2* and *MAP2K7*, a further significant decrease in the concentration was observed during the observation period (Table 5; *p* < 0.05).

**Table 5.** Concentration of MAP kinase-related genes at the protein level in HaCaT culture exposed to LPS and CsA.

Protein	C [ng/mL]	LPS [ng/mL]	H-2 [ng/mL]	H-8 [ng/mL]	H-24 [ng/mL]	*p*-value Student’s t-test or ANOVA	*p*-value Tukey’s post-hoc test
*DUSP1*	0.92 ± 0.09	0.32 ± 0.11	below the detection threshold	below the detection threshold	below the detection threshold	<0.001^a^	-
*DUSP4*	1.06 ± 0.11	0.56 ± 0.01	below the detection threshold	below the detection threshold	below the detection threshold	<0.001^a^	-
*MAP2K2*	2.11 ± 0.12	1.87 ± 0.34	0.34 ± 0.09	0.21 ± 0.12	0.20 ± 0.02	<0.001^b^	<0.001^1,2,3,4,5,6,7^ 1^10^ 0.004^8,9^
*MAP2K7*	1.79 ± 0.19	1.56 ± 0.12	0.87 ± 0.03	0.81 ± 0.09	0.80 ± 0.12	<0.001^b^	<0.001^1,2,3,4,5,6,7^ 1^10^ 0.004^8,9^
*MAP3K2*	0.98 ± 0.07	0.54 ± 0.05	below the detection threshold	below the detection threshold	below the detection threshold	<0.001^a^	-
*MAPK9*	4.12 ± 0.78	2.19 ± 0.34	below the detection threshold	below the detection threshold	below the detection threshold	<0.001^a^	-

LPS – lipopolysaccharide A; C – control culture; H_2, H_8, H_24 – culture exposed to cyclosporine A for 2, 8, 24 hours; DUSP1 – dual specificity phosphatase 1; DUSP4 – dual specificity phosphatase 4; MAP2K2 – mitogen-activated protein kinase kinase 2; MAP2K7 – mitogen-activated protein kinase kinase 7; MAP3K2 – mitogen-activated protein kinase kinase kinase 2; MAPK9 – mitogen-activated protein kinase 9; statistically significant differences between: ^1^C vs. LPS; ^2^C vs. H_2; ^3^C vs. H_8; ^4^ C vs. H_24; ^5^LPS vs. H_2; ^6^LPS vs. H_8; ^7^LPS vs. H_24; ^8^H_2 vs. H_8; ^9^H_2 vs. H_24; ^10^H_8 vs. H_24.

## Discussion

Psoriasis is an autoimmune disease with a multifactorial inheritance model [28–31]. Environmental and genetic factors are involved in the development of this disease [28–31]. Determining the expression of genes underlying the development of psoriasis is a huge challenge due to the phenotypic heterogeneity of this disease [28–31]. The true cause of the disease and the molecular mechanisms driving the development of psoriasis are still not fully understood [28–31]. Currently, complete cure of psoriasis is not possible, and the goal of treatment is to maintain remission as long as possible [28–31]. The methods of treating moderate and severe psoriasis are biological drugs, which improve the clinical condition of most patients, increasing the quality of life and enabling everyday functioning [32–34]. The limitations of biological treatment are the side effects of drugs and the loss of cell sensitivity to the drug used [32–34].

The MAP kinase family is activated in a cascade and transmits signals as a cellular response to external signals. Many intracellular processes occur under their control, including gene transcription, protein biosynthesis, cell division, differentiation, and survival or apoptosis [2,35]. The undisturbed operation of these pathways is therefore reflected in the proper functioning of the body [2,35]. Disruptions in MAP kinase pathways play a key role in the course of psoriasis, influencing the induction and development of the inflammatory process, regulating the synthesis of pro-inflammatory cytokines and influencing the expression of genes encoding proteins that participate in the transmission of these signals [2]. It should be noted that research is already being carried out on blocking MAPK-dependent signaling pathways at various levels of the signaling cascade in different diseases, including rheumatoid arthritis [36], melanoma [6], neuroblastoma [12], Alzheimer’s disease [37], cardiovascular disease [38].

Krawczyk et al. assessed the transcriptional activity of MAPK pathway genes and noticed changes in the expression level of genes related to apoptosis in patients with psoriatic arthritis who were treated with adalimumab [39]. They showed a decrease in MAP3K1 gene expression in patients before and during adalimumab therapy [39]. Interestingly, they used the HG-U133A 2.0 oligonucleotide microarray technique, which is the same as in our study [39]. Tan et al. showed a statistically significant decrease in the expression of the MAPK group genes using sequencing [40]. They demonstrated the significant involvement of these genes in the response to pro-inflammatory cytokines in psoriasis and rheumatoid arthritis [40].

DUSP phosphatases, which constitute a subgroup of MAPKs, are involved in the dephosphorylation of many signaling molecules, including MAPK, which affects their activity [41]. Kjellerup et al. obtained a total RNA from cultures of normal epidermal keratinocytes stimulated with pro-inflammatory cytokines and psoriatic skin [42]. They showed that the change in *DUSP1* expression is significantly reduced in psoriatic skin lesions compared to normal skin. They have reported that reducing *DUSP1* levels may contribute to chronic inflammation in psoriasis [42].

The work of Krawczyk et al. [39] and Kjellerup et al. [42] became the basis for our research, in which we assessed changes in the expression of MAPKs pathway genes under the influence of LPS and CsA in a human keratinocyte cell-line HaCaT [39,42]. We noticed changes in the expression profile of 6 mRNAs: *DUSP1*, *DUSP4*, *MAP2K2*, *MAP2K7*, *MAP3K2* and *MAPK9* in the HaCaT cell culture exposed to LPS and CsA compared to the control culture. Under the influence of LPS added to keratinocyte cultures, the expression of all genes was silenced. However, when CsA was added to the culture, an increase in the transcriptional activity of all genes except *DUSP4* was observed. In the next step, we demonstrated that miR-34a potentially regulates the activity of *DUSP1* and *DUSP4*; miR-1275 is involved in the regulation of *MAPK9* expression; miR-382 and miR-3188 potentially regulate *DUSP4* levels; and mir-200-5p is involved in the regulation of *MAP2K7* and *MAP3K2* expression. *MAP2K2* expression is most likely not regulated by the selected miRNAs. Analysis at the protein level revealed reduced concentrations of all proteins in the culture with LPS compared to the control. In turn, after adding CsA to the culture, the levels of DUSP1, DUSP4, MAP3K2 and MAPK9 were below the detection level, and the expression of MAP2K2, MAP2K7 was decreased.

Therefore, our analysis indicates an increase in the *DUSP1* mRNA level and a decrease in protein concentration after adding CsA to the culture, which may be due to the potential involvement of miRNAs. The identified miR-34a showed increased activity in HaCaT cells with LPS compared to the control. Interestingly, miR-34a is considered a potential therapeutic target in psoriasis [43]. Chen et al. observed that overexpression of this miRNA inhibits the proliferation of HaCaT cells and additionally induces apoptosis [43]. Moreover, Wang et al. reported an increased level of miR-34a in the skin of patients with psoriasis vulgaris [44]. The association of *DUSP1* and miR-34a has previously appeared in relation to osteosarcoma [45] and pulmonary artery smooth muscle cells [46].

Moreover, miR-34a may also be potentially involved in the regulation of *DUSP4* activity. Parveen et al. showed that *DUSP4* silencing promotes the generation of an anti-inflammatory response [47]. In turn, Cornell et al. observed that downregulation of this gene in mice improved survival in cases of sepsis [48]. On the other hand, Dougherty et al. showed that overexpression of *DUSP4* reduces oxidative stress [49]. Similarly, Li et al. reported a reduced level of *DUSP4* in osteoarthritis, and its increase inhibited the activation of the MAPK pathway, reducing oxidative stress, apoptosis and the inflammatory response [50]. In our study, *DUSP4* expression increased after CsA administration, then decreased after 8 hours of exposure, and began to increase again. The analysis showed that, in addition to miR-34a, miR-3188 and miR-382 may also be involved in the regulation of the activity of this gene. The expression of miR-382 was lower in the culture with the addition of CsA compared to the culture with LPS alone. Wang et al. showed that lower levels of this miRNA were important for the suppression of inflammation [51]. The observed significant decrease in *DUSP4* level after 8 hours of exposure to CsA may be related to the increase in miR-3188 activity, which reached a level higher than in culture with LPS alone. Li et al. showed that overexpression of miR-3188 resulted in reduced production of inflammatory cytokines in the case of atherosclerosis [52].

In our study, we also observed a potential effect of miR-200a-5p on the expression of *MAP2K7* and *MAP3K2*. Dolcino et al. demonstrated increased *MAP2K7* expression in patients with ankylosing spondylitis [53]. In turn, Huang et al. observed high levels of *MAP3K2* in cases of acute myocardial infarction. Overexpression of miR-1184, which participates in the regulation of *MAP3K2* activity, alleviated hypoxia-induced injury by MAP3K2 [54]. Importantly, Magenta et al. observed increased miR-200a levels in psoriasis patients [55], which is consistent with our observations. We noted its increased activity in the culture of HaCaT with LPS and its decrease only after the addition of CsA. It is possible that the increased expression of these genes in the culture with the addition of CsA is related to the reduced level of miR-200a. Low or undetectable protein concentrations may indicate additional mechanisms of posttranscriptional regulation, including miRNAs, that were not identified in this study.

In the present study, we also reported the potential association of *MAPK9* and miR-1275. Denninger et al. showed that reduced *MAPK9* levels exacerbated the clinical symptoms of the inflammatory response [56]. In addition, Yang et al. observed exacerbation of lung inflammation and injury during sepsis and acute lung injury in mice with low MAPK9 levels [57]. Interestingly, miR-1275 is the subject of many studies. They indicate that it participates in the regulation of many signaling pathways, including MAPK, ERK/JNK, Wnt, as well as phosphoinositide 3-kinase (PI3K)/protein kinase B (AKT). This gene is particularly studied in cancer, where, depending on its type, *MAPK9* may act as both a tumor suppressor and inducer [58]. Studies have also shown low levels of MAPK9 in COVID-19 patients [59,60], suggesting its role in the induction and development of the inflammatory process regardless of the type of disease. Our study revealed an increased level of miR-1275 in HaCaT culture after the addition of CsA compared to culture with LPS alone. This could potentially affect the level of *MAPK9* protein, which was below the detection threshold in the culture exposed to CsA.

In summary, this study allowed us to select MAPK-related genes whose expression changes in keratinocytes exposed to LPS and CsA, which may be useful in psoriasis research. We managed to predict which miRNAs may be involved in regulating the activity of these genes and thus become a potential marker or therapeutic target.

Of course, our research has limitations. First, the analysis was performed only on one cell line, *in* vitro model. Second, miRNA prediction is based on a bioinformatics database and is not experimentally confirmed. Third, we evaluated the influence of only one pro-inflammatory agent and drug used in the psoriasis vulgaris treatment.

Nevertheless, we demonstrated a potential relationship between *DUSP1* and miR-34a; *DUSP4* and miR-34a, miR-382, and miR-3188; *MAPK9* and miR-1275, *MAP2K7* and mir-200-5p; *MAP3K2* and mir-200-5p, which may be the subject of further research in the context of psoriasis. The discovery of new markers could help detect diseases without the full manifestation of symptoms and monitor the response to treatment [7,61–63].

Therefore, a pertinent and warranted next stage of research would involve evaluating the expression of MAPKs-related genes and miRNAs regulating their expression in other cell lines, including normal keratinocytes, or in co-culture with leukocytes. Additionally, it is justified to examine changes in the mRNA and miRNA patterns identified in our study in clinical materials such as whole blood and serum from patients using CsA for Psoriasis vulgaris. Finally, in the subsequent phase, we intend to assess the impact of other drugs used in psoriasis on the expression of MAPKs-related genes and miRNAs regulating their expression, while also determining the correlation between their expression and clinical indicators of the disease’s progression

## Data Availability

All data were included in the paper.
